# Shortening of apparent transverse relaxation time of inorganic phosphate as a breast cancer biomarker

**DOI:** 10.1002/nbm.4011

**Published:** 2018-10-12

**Authors:** Wybe J.M. van der Kemp, Tijl A. van der Velden, Alexander M. Schmitz, Kenneth G. Gilhuijs, Peter R. Luijten, Dennis W.J. Klomp, Jannie P. Wijnen

**Affiliations:** ^1^ Department of Radiology University Medical Center Utrecht Utrecht The Netherlands

**Keywords:** ^31^P, 7 T, breast cancer, glycolysis, MRSI, transverse relaxation time

## Abstract

Phosphorus MRS offers a non‐invasive tool for monitoring cell energy and phospholipid metabolism and can be of additional value in diagnosing cancer and monitoring cancer therapy. In this study, we determined the transverse relaxation times of a number of phosphorous metabolites in a group of breast cancer patients by adiabatic multi‐echo spectroscopic imaging at 7 T. The transverse relaxation times of phosphoethanolamine, phosphocholine, inorganic phosphate (P_i_), glycerophosphocholine and glycerophosphatidylcholine were 184 ± 8 ms, 203 ± 17 ms, 87 ± 8 ms, 240 ± 56 ms and 20 ± 10 ms, respectively. The transverse relaxation time of P_i_ in breast cancer tissue was less than half that of healthy fibroglandular tissue. This effect is most likely caused by an up‐regulation of glycolysis in breast cancer tissue that leads to interaction of P_i_ with the GAPDH enzyme, which forms part of the reversible pathway of exchange of P_i_ with gamma‐adenosine tri‐phosphate, thus shortening its apparent transverse relaxation time. As healthy breast tissue shows very little glycolytic activity, the apparent *T*_2_ shortening of P_i_ due to malignant transformation could possibly be used as a biomarker for cancer.

## Graphical abstract

Transverse relaxation times of phosphorous metabolites were determined in breast cancer tissue at 7 T. The apparent *T*_2_ of P_i_ was less than half that of healthy fibroglandular tissue. This effect is most likely caused by an up‐regulation of glycolysis in breast cancer tissue that leads to increased interaction of P_i_ with the GADPH enzyme, thus shortening its apparent *T*_2_. As healthy breast tissue shows very little glycolytic activity, the observed effect may serve as a biomarker for cancer.

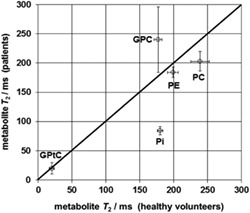

**Abbreviations**
1,3‐DPG: 1,3‐diphosphoglycerate
AMESING: adiabatic multi‐echo spectroscopic imaging
ATP: adenosine tri‐phosphate
EDTA: ethylenediaminetetraacetic acid
GAPDH: glyceraldehyde‐3‐phosphate dehydrogenase
GPC: glycerophosphocholine
GPE: glycerophosphoethanolamine
GPtC: (diacyl‐)glycerophosphatidylcholine
GPtE: (diacyl‐)glycerophosphatidylethanolamine
MRSI: magnetic resonance spectroscopic imaging
PC: phosphocholine
PCr: phosphocreatine
PDE: phosphodiester
PE: phosphoethanolamine
PGK: phosphoglycerate kinase
P_i_: inorganic phosphate
PME: phosphomonoester

## 1 INTRODUCTION

Phosphorus magnetic resonance spectroscopic imaging (^31^P MRSI) enables non‐invasive measurement of phospholipid membrane metabolism and cell energy metabolism in vivo. It is well established that enhanced levels of phosphomonoesters (PMEs)—phosphocholine (PC) and phosphoethanolamine (PE)—are a metabolic hallmark of cancer,^1^ because these metabolites are key intermediates in phospholipid synthesis, which is enhanced in cancer. Metabolite ratios of PMEs to phosphodiesters (PDEs, namely glycerophosphocholine (GPC) and glycerophosphoethanolamine (GPE),^2^ PMEs to inorganic phosphate (P_i_)^3^, ^4^ and PMEs to gamma‐adenosine tri‐phosphate (γ‐ATP)^2^ have been proposed as biomarkers to assess response to cancer therapy.

**Figure 1:**
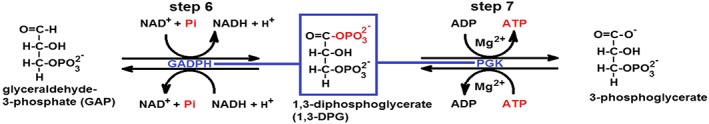
The reversible Steps 6 and 7 of the glycolytic pathway involving P_i_. The reaction intermediate 1,3‐DPG is channelled between the GAPDH and PGK enzymes, without being released into the aqueous environment^7^, ^8^

Another metabolic hallmark of cancer is up‐regulated aerobic glycolysis,^5^ also known as the Warburg effect. Within the glycolytic pathway Steps 6 and 7 involve P_i_ and these steps are fully reversible^6^ reactions (Figure 1). In Step 6, catalyzed by the enzyme glyceraldehyde‐3‐phosphate dehydrogenase (GAPDH), glyceraldehyde‐3‐phosphate is converted into 1,3‐diphosphoglycerate (1,3‐DPG) by attaching P_i_. In Step 7, the reaction product 1,3‐DPG is converted to 3‐phosphoglycerate under the influence of phosphoglycerate kinase (PGK). Additionally, the phosphate group is transferred to ADP in Step 7. From kinetic modelling^7^ and fluorescence energy transfer measurements,^8^ it has become evident that the enzymes GAPDH and PGK form a loosely bound complex and the intermediate metabolite 1,3‐DPG is directly transferred from one enzyme to the next without being released into the aqueous environment.

Effectively, P_i_ is reversibly exchanged with γ‐ATP via this two‐step process, which can lead to a substantial line broadening of the P_i_ signal, i.e. a shortening of its apparent transverse relaxation time, as the maximum enzymatic capacities of GAPDH and PGK can be several hundreds of millimoles per minute per kilogram of tissue, depending on the tissue at hand.^9^

While in vivo metabolite ratios measured by MRS should be interpreted carefully, because of their dependence on the combination of flip‐angle, repetition time and intrinsic longitudinal relaxation time of the metabolites, measured apparent *T*_2_ values of metabolites can be compared readily and can provide information about the mobility of metabolites. It is well known, for instance, that the large PDE peaks observed at low field in the human breast are mainly from mobile phospholipids and not from aqueous GPC and GPE. However, also at 7 T, mobile phospholipids are the main contribution to the PDE signal in the breast as based on the short apparent *T*_2_ of these signals.^10^

Here we show in a group of breast cancer patients an apparent shortening of the transverse relaxation time of the P_i_ signal, as compared with fibroglandular breast tissue of healthy volunteers, which could well be caused by a mobility restriction on P_i_ by enzymatic interaction with GAPDH. Additionally, to rule out pH dependence or the effect of general P_i_‐protein interactions on the transverse relaxation time of P_i_, we quantify these influences on the *T*_2_ of P_i_ solutions in phantom measurements.

## 2 METHODS

### 2.1 In vivo measurements

A group of 24 patients (age range 47–71; average age 59) with breast cancer (identified by the hyperintense area of the dynamic contrast enhanced MRI series and confirmed by histopathology of biopsy from the lesion) comprising 28 lesions (lesion size ≤2 cm; 26 invasive ductal carcinomas, 2 invasive lobular carcinomas) were scanned on a whole‐body 7 T MR system (Philips, Best, The Netherlands) with a dual tuned unilateral breast coil. The scan protocol consisted of basic imaging and ^31^P MRSI with an adiabatic multi‐echo sequence (AMESING) that was implemented on the scanner as a software patch that has been described in detail elsewhere,^11^ with *T*_R_ = 6 s, Δ*T*_E_ = 45 ms, matrix 8 × 8 × 8, field of view 160 × 160 × 160 mm^3^, acquiring one FID and five full echoes with spherical *k*‐space sampling, BW = 8200 Hz and 256 data points for the echoes. The pulse durations of the adiabatic half passage (AHP) and the BIR‐4 refocusing pulses that were used in the multi‐echo sequence were 2 and 8 ms, respectively; the applied power level was *γB*_1_ = 1700 Hz. The center frequency of the pulses was at 4.2 ppm, in between the PDE and PME resonances. At the applied power level the 95% excitation bandwidth of the AHP pulse is 12 ppm and the 95% refocused component of the BIR‐4 180 pulse is 9 ppm, causing a maximum of 5% modulation in odd‐even echoes that should not affect the *T*_2_ calculation. The TR is 6 s, which is close to the optimal for PMEs, PDEs and P_i_ (optimal *T*_R_ = 1.25 *T*_1_). The total scan time of the ^31^P MRSI acquisition was 25 min with one sample average. The study was approved by the medical ethical committee of the University Medical Centre Utrecht and all patients gave written informed consent to participate in the study.

### 2.2 Phantom measurements

Six 100 mL phantoms containing 20mM NaH_2_PO_4_/Na_2_HPO_4_ in 140mM NaCl and 0.4mM Na_2_EDTA in the pH range 6.5 to 7.5 were measured with the same adiabatic multi‐echo sequence as was used for the patients. The complexing agent ethylenediaminetetraacetic acid (EDTA) was added to the solutions to minimize the effect of trace metal contamination on the line width of P_i_. After the phantoms were measured with ^31^P MRSI, egg albumen (fresh egg white) was added to the solutions amounting to 5 vol.%, and after measurement of the pH (MeterLab PHM210, Radiometer, Copenhagen, Denmark) the albumen P_i_ solutions were measured again with ^31^P MRSI. The solutions remained clear and stable over the course of the experiments.

### 2.3 Analysis of in vivo data

Acquired ^31^P MRSI data were processed with home built IDL software. Data were spatially Hanning filtered, apodized (10 Hz Lorentzian) and zero filled to 2048 data points. Spectra were zeroth‐ and first‐order phased and the baselines of the FID spectra were corrected by fitting a second‐order spline. Per subject, data from one or more voxels encompassing the tumor(s) were taken. The spectra (FID and echoes) of the different patients were referenced and aligned to the α‐ATP of the FID spectrum and summed to patient group data leading to one summed FID spectrum and five summed echo spectra. All tumor sizes were smaller than the nominal voxel size, and standard voxel shifting (over *x*, *y* or *z* performed in *k*‐space by a linear change of phase on *k*_*x*_, *k*_*y*_ or *k*_*z*_ respectively) was used to select the tumor volume within one voxel. The group data were spectrally fitted in JMRUI^12^ using the AMARES^13^ algorithm with the following a priori constraints: 0.50 ppm chemical shift difference between PE and PC, and 0.56 ppm chemical shift difference between (glycerophosphatidylethanolamine (GPtE) + GPC) and glycerophosphatidylcholine (GPtC).^14^ Line widths for PE, PC and P_i_ were free but identical and line widths for γ‐NTP and α‐NTP were also free but identical. *T*_2_ fits of the different ^31^P metabolites were made and breast cancer ^31^P spectra and metabolite *T*_2_ values were compared with spectra and metabolite *T*_2_ values of healthy fibroglandular tissue, which were determined in a previous study.^14^

### 2.4 Analysis of phantom data

Acquired ^31^P MRSI data were Hanning filtered, apodized (10 Hz Lorentzian) and zero filled to 2048 data points. The FID and echo spectra of individual measurements at the various pH values were fitted in JMRUI^12^ using AMARES^13^ algorithms and the *T*_2_ of P_i_ was fitted mono‐exponentially.

## 3 RESULTS

**Figure 2:**
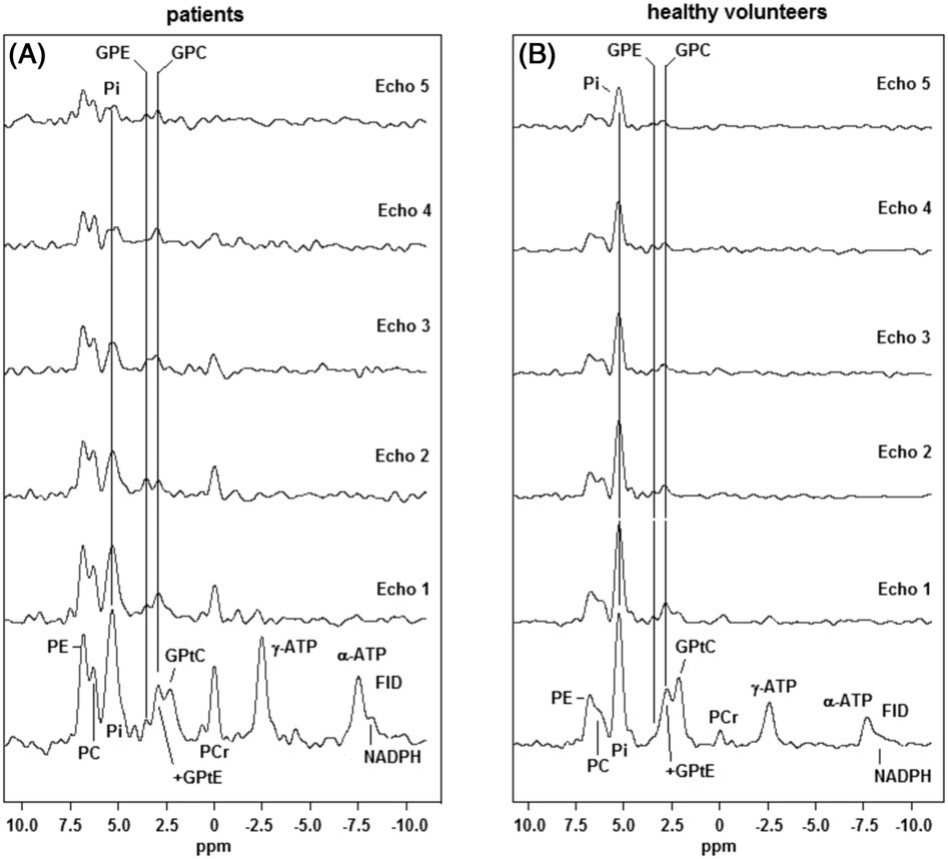
Summed FID and echo ^31^P MR spectra of 28 breast cancer lesions of a group of 24 patients (A) and of fibroglandular breast tissue of 8 healthy volunteers (B).^14^ Note that the P_i_ signal in the breast cancer spectrum drops below the PME signals in the later echoes, while in the healthy spectrum it remains the highest signal throughout all echoes

The FID spectra and the echo spectra of the patient group and the healthy volunteer group^14^ are shown in Figure 2A and 2B respectively. The spectra are scaled to the same intensity of P_i_ in the FID. Note the high PE and PC signals as compared with P_i_ and PDEs in the breast cancer spectra, while in the healthy spectra PE and PC are lower relative to these signals. Also note the high PDE signals around 2.5 ppm in the summed FID spectrum that drops in intensity with the first echo time of 45 ms (breast cancer as well as healthy tissue). The phosphocreatine (PCr) peak intensity at 0 ppm is somewhat higher in the patient spectra than in the healthy volunteer spectra, because on average the tumor voxels are closer to the pectoralis muscle, with high PCr intensity, than the voxels that were chosen in the healthy volunteers, where we tried to minimize this contamination.

**Figure 3:**
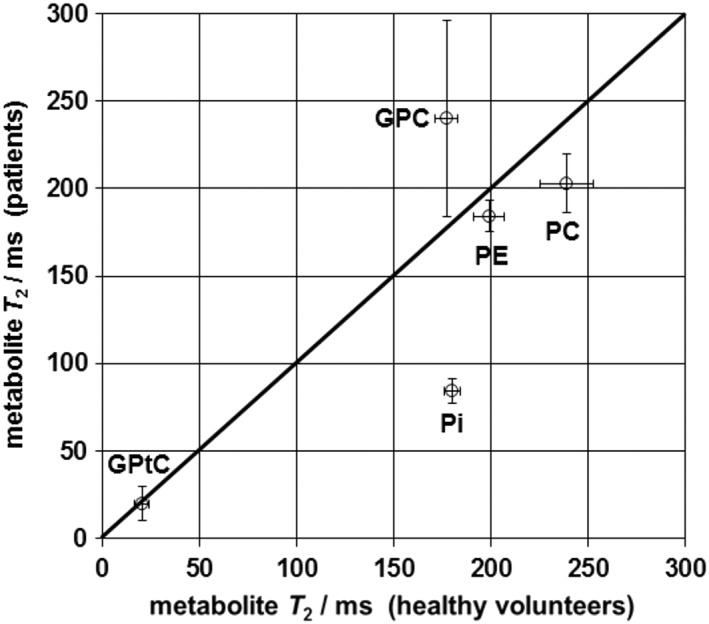
Fitted *T*_2_ values of some selected ^31^P metabolites in breast cancer tissue versus fitted values in healthy fibroglandular tissue. Overall, the *T*_2_ values of the ^31^P metabolites in breast cancer tissue line up well with those in healthy volunteers, obtained previously,^14^ apart from P_i_

In the spectra of healthy tissue the P_i_ signal is by far the highest and this remains the case also in the echoes, while in the breast cancer spectra the signal intensity of P_i_ drops even below the PME signal intensities. Transverse relaxation times (±sd obtained from *T*_2_ fit) of PE, PC, P_i_, GPC and GPtC that were fitted for breast cancer tissue were 184 ± 8 ms, 203 ± 17 ms, 87 ± 8 ms, 240 ± 56 ms and 20 ± 10 ms, respectively. Figure 3 shows fitted *T*_2_ values of various ^31^P metabolites in breast cancer tissue as a function of those in healthy fibroglandular tissue. There is a substantial difference in the *T*_2_ values of P_i_, which are more than twice as long in healthy volunteers as in patients.

**Figure 4:**
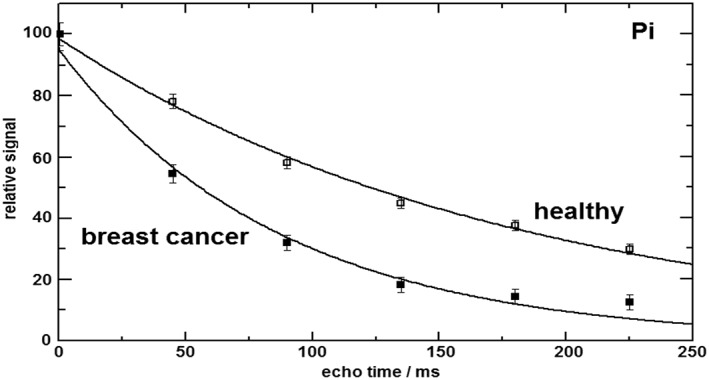
Fitted transverse relaxation curves of P_i_ in breast cancer tissue, *T*_2_ = 87 ± 8 ms, and healthy fibroglandular breast tissue, *T*_2_ = 180 ± 4 ms

Figure 4 shows a direct comparison of the *T*_2_ fits for P_i_ in breast cancer tissue and healthy fibroglandular tissue. Breast cancer tissue shows a transverse relaxation time for P_i_ of 87 ± 8 ms, whereas the value in healthy fibroglandular breast tissue is 180 ± 4 ms. The error bars in Figure 4 are based on the quality of the spectral fitting (Cramer‐Rao bounds) with AMARES of the (FID and echo) P_i_ signals.

**Figure 5:**
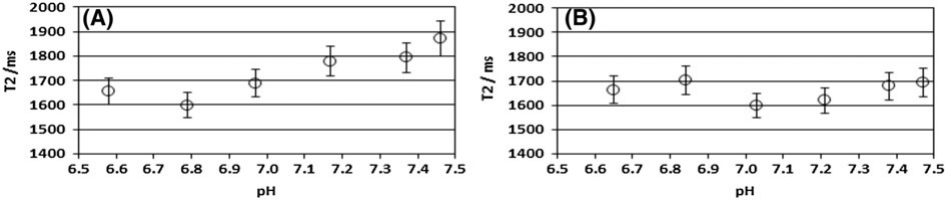
A, Transverse relaxation time (±sd of fit) of P_i_ in solutions of 20mM sodium phosphate (in 140mM NaCl; 0.4mM Na_2_EDTA) as a function of pH. B, As in A with 5% fresh egg albumen added to the solutions. The complexing agent EDTA was added to minimize the influence of paramagnetic trace metals. Note the order of magnitude longer *T*_2_ for P_i_ and the relative insensitivity of *T*_2_ to pH

The influence of pH and added protein (egg albumen) on the *T*_2_ of in vitro P_i_ solutions is shown in Figure 5A and 5B, respectively.

As can be seen from Figure 5A and 5B, only a small increase (~15%) in *T*_2_ is observed when pH increases from 6.8 to 7.45. Addition of protein tends to dampen this effect.

## 4 DISCUSSION

The breast cancer spectra of Figure 2A clearly show higher PME to P_i_ and PME to PDE ratios as compared with the spectra from healthy fibroglandular breast tissue, indicative of enhanced phospholipid synthesis. The PDE metabolites in these spectra (breast cancer as well as healthy tissue) display a short *T*_2_ component that we subscribe to mobile phospholipids^10^ in the form of GPtC and GPtE, as these signals disappear in the noise after the second echo. The aqueous PDE signals GPC and GPE remain visible at longer echo times. Patient spectra show a somewhat larger PCr signal than spectra from healthy volunteers. The PCr signal is due to voxel bleeding from the pectoralis muscle. On average, patient tumor voxels are located closer to the pectoralis muscle than the voxels chosen in the healthy volunteers that were closest to the nipple. The P_i_ signal in the breast cancer spectra decreases faster with echo time than the P_i_ signal in the spectra of healthy breast tissue. In fact, the apparent *T*_2_ of P_i_ in breast cancer tissue is less than half that in healthy breast tissue, as can be observed from Figures 3 and 4, while the apparent *T*_2_ of the other ^31^P metabolites does not differ significantly between breast cancer and healthy tissue. As tumor tissue often shows a lower pH than healthy tissue, one may suspect the halved *T*_2_ of P_i_ in breast cancer to be pH related. However, the phantom measurements of *T*_2_ (Figure 5A and 5B) of P_i_ indicate that the shorter *T*_2_ of Pi in breast cancer tissue is probably *not* caused by a general pH effect on the *T*_2_ of P_i_ and/or a pH dependent general Pi_−_protein interaction, as the influence of pH and general protein interaction on the *T*_2_ of P_i_ is only minor (Figure 5A and 5B). A possible mechanism for the apparent *T*_2_ shortening effect of P_i_ in breast cancer tissue could be up‐regulated glycolysis. This leads to the exchange of P_i_ with γ‐ATP via the fully reversible Steps 6 and 7 of the glycolytic pathway as shown in Figure 1. It should be noted here that the exchange of P_i_ with γ‐ATP is too slow to be of significance for shortening the *T*_2_ of P_i_. For instance, the exchange rate of P_i_ ↔ γ‐ATP in the brain is 0.2 s^−1^,^15^, ^16^ and in resting muscle it is 0.06 s^−1^.^17^ It is the reversible interaction with the GAPDH enzyme, which forms part of the pathway of P_i_ exchange with γ‐ATP (Figure 1), that causes the apparent *T*_2_ shortening of P_i_. Although the exchange from P_i_ to γ‐ATP and vice versa is relatively slow, the reversible interaction of P_i_ with GAPDH can be very fast (the maximum enzymatic capacity of GAPDH can be several hundred millimoles per minute depending on the tissue). Only a small fraction of the interactions of P_i_ with the GAPDH enzyme leads to the actual formation of ATP via this pathway. Likewise, only a small fraction of the interactions of ATP with the PGK enzyme leads to the formation of 1,3‐DPG and subsequently to P_i_ via GAPDH.

When the exchange of P_i_ with GAPDH is faster than the transverse relaxation of P_i_, this will lead to a reduction of signal over the echoes and thereby leads to an apparently lower *T*_2_ value.

The measured apparent *T*_2_ of P_i_ is an average value over intracellular and extracellular compartments, which are difficult to distinguish based on a subtle chemical shift difference (best viewed in final echo where signal levels seem comparable). Higher fields, or better *B*_0_ shimming, may distinguish these P_i_ compartments better, yet the GAPDH enzyme is found intracellularly as well as extracellularly^6^ and can thus affect the apparent *T*_2_ of P_i_ in both compartments. Up‐regulated glycolysis is corroborated by the average GAPDH gene expression in breast cancer, which is over four times higher than in normal breast tissue.^18^ Song et al. even reported an increase in GAPDH gene expression of a factor of 5 to 21.^19^ Enhanced expression of GAPDH is associated with breast cancer cell proliferation and tumor aggressiveness.^20^

Notably, glycolytic conversion of glucose is also important in muscle metabolism at rest^17^ and in the brain, where short transverse relaxation times for P_i_ are encountered as well. Muscle and brain tissue are among the tissues with the highest GAPDH mRNA expression, while healthy breast tissue has the lowest expression.^21^ The reported *T*_2_ of P_i_ in muscle at rest at 7 T is around 100 ms (comparable to the *T*_2_ value of P_i_ in breast cancer tissue), which is substantially shorter than the *T*_2_ values for PCr and the PDEs in muscle.^22^ Recently, we measured the transverse relaxation times of the PMEs, PDEs and P_i_ in the healthy human brain at 7 T. In that study we also found a short relaxation time of P_i_, only 86 ± 2 ms.^23^

The present study has several limitations. The breast cancerous lesions were all relatively small and therefore SNR of the spectra of the individual patients was too low to carry out *T*_2_ fitting of metabolites. To enhance SNR and the reliability of *T*_2_ fitting of ^31^P metabolites, the breast cancer spectra of the patient group were averaged. To reduce voxel bleeding, Hanning filtering was performed before analysis, which increases the nominal voxel size by a factor of 1.78 in all spatial dimensions.^24^ Although the voxels for which spectra were analyzed were carefully positioned around the cancerous lesions, some partial volume effect with healthy tissue is inevitable. However, with the proposed *T*_2_ shortening mechanism of P_i_, a mixture of cancerous and healthy fibroglandular tissue would lead to an increase in *T*_2_ of P_i_ with increasing healthy tissue, contrary to the effect that we have measured. Considering the large voxel size and the relatively low density of glandular breast tissue in these older women, it was not possible to do an analysis of healthy glandular tissue with the unilateral setup that was used. An analysis of the data on GAPDH expression from the human protein atlas^25^ in aging healthy fibroglandular breast tissue shows an average increase in GAPDH expression of 20% from women ranging between 25 and 65 years. Considering the difference in average age between the healthy volunteer group (26 years) and the patient group (59 years) it is to be expected that healthy breast tissue of the elderly patient group shows a shorter *T*_2_ of P_i_ than that of the younger healthy volunteer group. However, the increase in GAPDH expression due to ageing is only minor as compared with the increased GAPDH expression as a consequence of breast cancer, where an average increase of a factor of 4 has been reported.^18^

Definite proof for the proposed *T*_2_ shortening of P_i_ due to up‐regulated GAPDH expression (and glycolysis) could be obtained from comparing multi‐echo ^31^P MR spectra of knock down GAPDH breast cancer xenografts with non‐modified breast cancer xenografts.

In conclusion, the apparent transverse relaxation time of P_i_ in breast cancer tissue is less than half that in healthy fibroglandular tissue. The effect is most likely caused by an up‐regulation of GAPDH expression (and glycolysis) in breast cancer tissue that leads to fast interaction of P_i_ with GAPDH, which is also seen in muscle tissue and brain tissue, with high GAPDH expression and similar short *T*_2_ values for P_i_.

## FUNDING INFORMATION

This study was sponsored by Alpe d'Huzes UU2013–6302 and the Nederlandse Organisatie voor Wetenschappelijk Onderzoek VENI‐JW‐016.148.002.

## References

[1] Glunde K , Bhujwalla ZM , Ronen SM . Choline metabolism in malignant transformation. Nat Rev Cancer. 2011;11:835‐848. 22089420 10.1038/nrc3162 PMC4337883

[2] Podo F . Tumour phospholipid metabolism. NMR Biomed. 1999;12:413‐439. 10654290 10.1002/(sici)1099-1492(199911)12:7<413::aid-nbm587>3.0.co;2-u

[3] Sakurai H , Mitsuhashi N , Murata O , et al. Early radiation effects in highly apoptotic murine lymphoma xenografts monitored by ^31^P magnetic resonance spectroscopy. Int J Radiat Oncol Biol Phys. 1998;41:1157‐1162. 9719127 10.1016/s0360-3016(98)00158-8

[4] van der Kemp WJM , Stehouwer BL , Luijten PR , van den Bosch MAAJ , Klomp DWJ . Detection of alterations in membrane metabolism during neoadjuvant chemotherapy in patients with breast cancer using phosphorus magnetic resonance spectroscopy at 7 Tesla. SpringerPlus. 2014;3:634. 25932360 10.1186/2193-1801-3-634 PMC4409619

[5] Gatenby RA , Gillies RJ . Why do cancers have high aerobic glycolysis? Nat Rev Cancer. 2004;4:891‐899. 15516961 10.1038/nrc1478

[6] Seidler NW . GAPDH: Biological Properties and Diversity (Advances in Experimental Medicine and Biology 985). Springer; 2013.

[7] Weber JP , Bernhard SA . Transfer of 1,3‐diphosphoglycerate between glyceraldehyde‐3‐phosphatedehydrogenase and 3‐phosphoglycerate kinase via an enzyme‐substrate‐enzyme complex. Biochemistry. 1982;21:4189‐4194. 7126536 10.1021/bi00260a042

[8] Tomokuni Y , Goryo K , Katsura A , et al. Loose interaction between glyceraldehyde‐3‐phosphate dehydrogenase and phosphoglycerate kinase revealed by fluorescence resonance energy transfer‐fluorescence lifetime imaging microscopy in living cells. FEBS J. 2010;277:1310‐1318. 20392205 10.1111/j.1742-4658.2010.07561.x

[9] Scrutton MC , Utter MF . The regulation of glycolysis and gluconeogenesis in animal tissues. Annu Rev Biochem. 1968;37:249‐302.

[10] van der Kemp WJ , Stehouwer BL , Runge JH , et al. Glycerophosphocholine and glycerophosphoethanolamine are not the main sources of the in vivo ^31^P MRS phosphodiester signals from healthy fibroglandular breast tissue at 7 T. Front Oncol. 2016;6:29. 26913240 10.3389/fonc.2016.00029 PMC4753293

[11] Van der Kemp WJM , Boer VO , Luijten PR , Stehouwer BL , Veldhuis WB , Klomp DW . Adiabatic multi‐echo ^31^P spectroscopic imaging (AMESING) at 7 T for the measurement of transverse relaxation times and regaining of sensitivity in tissues with short *T*₂ values. NMR Biomed. 2013;26:1299‐1307. 23553945 10.1002/nbm.2952

[12] Naressi A , Couturier C , Devos JM , et al. Java‐based graphical user interface for the MRUI quantitation package. Magn Reson Mater Phys Biol Med. 2001;12:141‐152. 10.1007/BF02668096 11390270

[13] Vanhamme L , van den Boogaart A , Van Huffel S . Improved method for accurate and efficient quantification of MRS data with use of prior knowledge. J Magn Reson. 1997;129:35‐43. 9405214 10.1006/jmre.1997.1244

[14] van der Kemp WJM , Stehouwer BL , Boer VO , Luijten PR , Klomp DWJ , Wijnen JP . Proton and phosphorus magnetic resonance spectroscopy of the healthy human breast at 7 T. NMR Biomed. 2017;30. 10.1002/nbm.3684 PMC5248643 28032377

[15] Ren J , Sherry AD , Malloy CR . Efficient ^31^P band inversion transfer approach for measuring creatine kinase activity, ATP synthesis, and molecular dynamics in the human brain at 7 T. Magn Reson Med. 2017;78:1657‐1666. 27868234 10.1002/mrm.26560 PMC5438784

[16] Ren J , Sherry AD , Malloy CR . ^31^P‐MRS of healthy human brain: ATP synthesis, metabolite concentrations, pH, and *T* _1_ relaxation times. NMR Biomed. 2015;28:1455‐1462. 26404723 10.1002/nbm.3384 PMC4772768

[17] Kemp GJ , Brindle KM . What do magnetic resonance‐based measurements of Pi→ATP flux tell us about skeletal muscle metabolism? Diabetes. 2012;61:1927‐1934. 22826313 10.2337/db11-1725 PMC3402329

[18] Isidoro A , Casado E , Redondo A , et al. Breast carcinomas fulfill the Warburg hypothesis and provide metabolic markers of cancer prognosis. Carcinogenesis. 2005;26:2095‐2104. 16033770 10.1093/carcin/bgi188

[19] Song MN , Moon PG , Lee JE , et al. Proteomic analysis of breast cancer tissues to identify biomarker candidates by gel‐assisted digestion and label‐free quantification methods using LC‐MS/MS. Arch Pharm Res. 2012;35:1839‐1847. 23139137 10.1007/s12272-012-1018-6

[20] Révillion F , Pawlowski V , Hornez L , Peyrat JP . Glyceraldehyde‐3‐phosphate dehydrogenase gene expression in human breast cancer. Eur J Cancer. 2000;36:1038‐1042. 10885609 10.1016/s0959-8049(00)00051-4

[21] Barber RD , Harmer DW , Coleman RA , Clark BJ . GAPDH as a housekeeping gene: analysis of GAPDH mRNA expression in a panel of 72 human tissues. Physiol Genomics. 2005;21:389‐395. 15769908 10.1152/physiolgenomics.00025.2005

[22] Bogner W , Chmelik M , Schmid AI , Moser E , Trattnig S , Gruber S . Assessment of ^31^P relaxation times in the human calf muscle: a comparison between 3 T and 7 T in vivo. Magn Reson Med. 2009;62:574‐582. 19526487 10.1002/mrm.22057

[23] van der Kemp WJM , Klomp DWJ , Wijnen JP . ^31^P transverse relaxation times of phosphomonoesters, phosphodiesters and inorganic phosphate in the human brain at 7 tesla. Magn Reson Med. 2018;80:29‐35. 29215148 10.1002/mrm.27026 PMC5900879

[24] Pohmann R , von Kienlin M . Accurate phosphorus metabolite images of the human heart by 3D acquisition‐weighted CSI. Magn Reson Med. 2001;45:817‐826. 11323808 10.1002/mrm.1110

[25] Uhlén M , Fagerberg L , Hallström BM , et al. Proteomics. Tissue‐based map of the human proteome. Science. 2015;347, 1260419. 25613900 10.1126/science.1260419

